# Pheochromocytoma and Behçet’s Disease: Exploring a Rare Coexistence

**DOI:** 10.7759/cureus.84812

**Published:** 2025-05-26

**Authors:** Niyas Khalid Ottu Para, Daya Mani Jacob, Divyashri Ramanathan Nagarajan, Diya E Viju, Anupama Kakade

**Affiliations:** 1 Internal Medicine, Burjeel Medical City, Abu Dhabi, ARE

**Keywords:** adrenal tumour, behcet’s disease, paragangliomas pheochromocytomas neuroendocrine tumor, pheochromocytoma, vasculitis

## Abstract

This case report describes a rare association between pheochromocytoma and Behçet's disease (BD) in a 38-year-old female. The patient presented with palpitations, dizziness, and blood pressure fluctuations, leading to the diagnosis of pheochromocytoma. Following treatment, she developed symptoms of BD, including oral and genital ulcers. This case highlights the challenges in the diagnosis of pheochromocytoma and raises questions about the potential link between catecholamine excess and autoimmune conditions like BD, suggesting a need for further studies into their pathophysiological relationship.

## Introduction

Pheochromocytomas are rare neuroendocrine tumors originating from the chromaffin cells of the adrenal medulla, cells that are derived from the neural crest. These tumors are characterized by the excessive secretion of catecholamines - primarily epinephrine and norepinephrine - leading to clinical symptoms such as hypertension, palpitations, headache, and diaphoresis. While most cases are sporadic, a notable proportion is linked to hereditary syndromes, including Multiple Endocrine Neoplasia type 2 (MEN2), Von Hippel-Lindau disease, Neurofibromatosis type 1, and familial paraganglioma syndromes. Genetic testing is recommended for all diagnosed patients, as a significant number of cases involve germline or somatic mutations, typically inherited in an autosomal dominant manner [1].

Pheochromocytomas are most commonly located in the adrenal glands, with a minority arising from extra-adrenal sites in the sympathetic ganglia, termed paragangliomas [2]. Their incidence ranges from 2 to 9.1 per million adults annually, and they account for up to 60% of adrenal incidentalomas. Though most are benign, 25% may be malignant. Diagnosis relies on biochemical tests measuring plasma or urinary metanephrines, with imaging modalities used for tumor localization and assessing potential metastasis. Surgical excision remains the definitive treatment, with preoperative alpha-adrenergic blockade to manage hypertension and arrhythmias [1].

Behçet’s disease (BD) is a chronic, multisystem inflammatory disorder characterized by recurrent oral and genital ulcers, ocular inflammation, and systemic manifestations, including vascular, neurological, and gastrointestinal (GI) involvement. Though the precise cause remains unclear, BD is considered an autoimmune vasculitis with a strong association to the HLA-B51 allele, particularly prevalent in regions along the ancient Silk Road [3].

This case report explores the rare coexistence of pheochromocytoma and BD - an inflammatory vasculitis with systemic involvement - highlighting diagnostic challenges, management considerations, and the need for multidisciplinary vigilance in complex presentations.

## Case presentation

A 38-year-old female presented with shortness of breath and dizziness, associated with epigastric pain and palpitations for five days. She had no fever, chest pain, or edema. She has a four-year history of episodic palpitations, dizziness, blood pressure fluctuations, irritability, anxiety, and agitation. Her past medical history was notable for bronchial asthma and a prior episode of deep vein thrombosis (DVT) in 2007. She denied any history of smoking or alcohol use. On examination, vital signs were within normal limits, except for persistent tachycardia, with a heart rate reaching 130 beats per minute.

**Figure 1:**
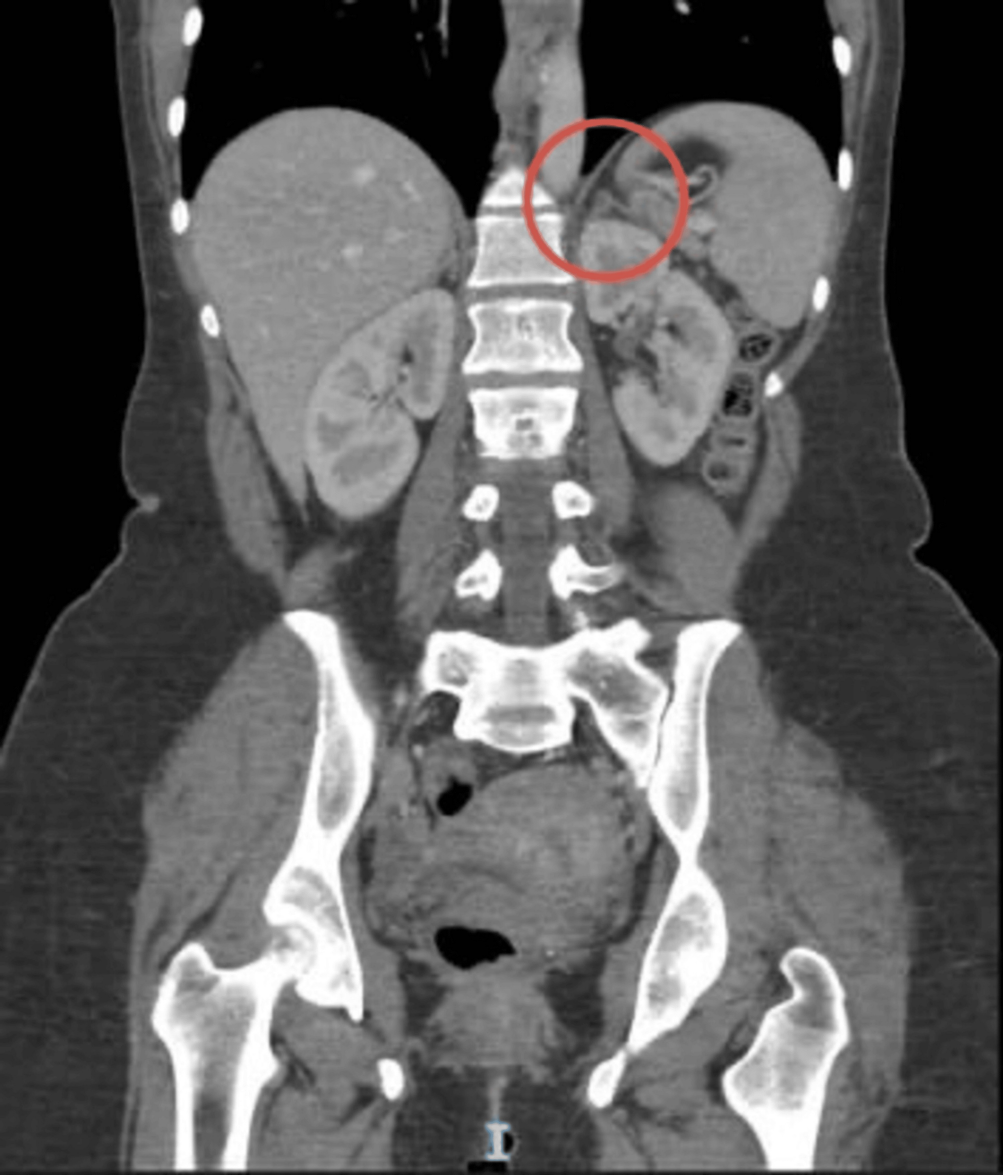
CT abdomen showing nodular thickening of the left adrenal gland (circled) CT, computed tomography

Extensive evaluations had been conducted in the United Arab Emirates and abroad, without a definitive diagnosis. Cardiac assessment, including Holter monitoring, revealed no significant arrhythmias. Given her constellation of symptoms, a differential diagnosis of pheochromocytoma was pursued. Laboratory testing demonstrated adrenaline of 13.8 µg/24 hr (<20 µg/24 hr), noradrenaline of 154.3 µg/24 hr (<90 µg/24 hr), and vanillylmandelic acid (VMA) of 10.6 mg/24 hr (<7 mg/24 hr). A contrast-enhanced computed tomography (CT) scan of the abdomen revealed a left adrenal adenoma (Figure 1). She was evaluated by GI surgery, and a dodecane tetraacetic acid positron emission tomography (DOTA PET) scan was advised but could not be done due to insurance-related issues. Her repeated urinary metanephrines and VMA were elevated from previous readings, and she continued to deteriorate symptomatically. She progressed to develop painful urinary tract infections (UTIs) with urinary retention.

She experienced recurrent admissions for abdominal pain and UTIs, during which her inflammatory markers were elevated, despite the absence of a mechanical obstruction on imaging. A urology consultation was obtained, and she was empirically initiated on an alpha-blocker; however, her urinary symptoms persisted. Subsequently, she developed refractory urinary retention, requiring catheterization. Repeat urine metanephrine levels were 990 µg/24 hr (reference range: 36-209 µg/24 hr), normetanephrine levels were 2247 µg/24 hr (131-612 µg/24 hr), and VMA was 10.6 mg/24 hr (<7 mg/24 hr), all of which were further elevated.

**Figure 2:**
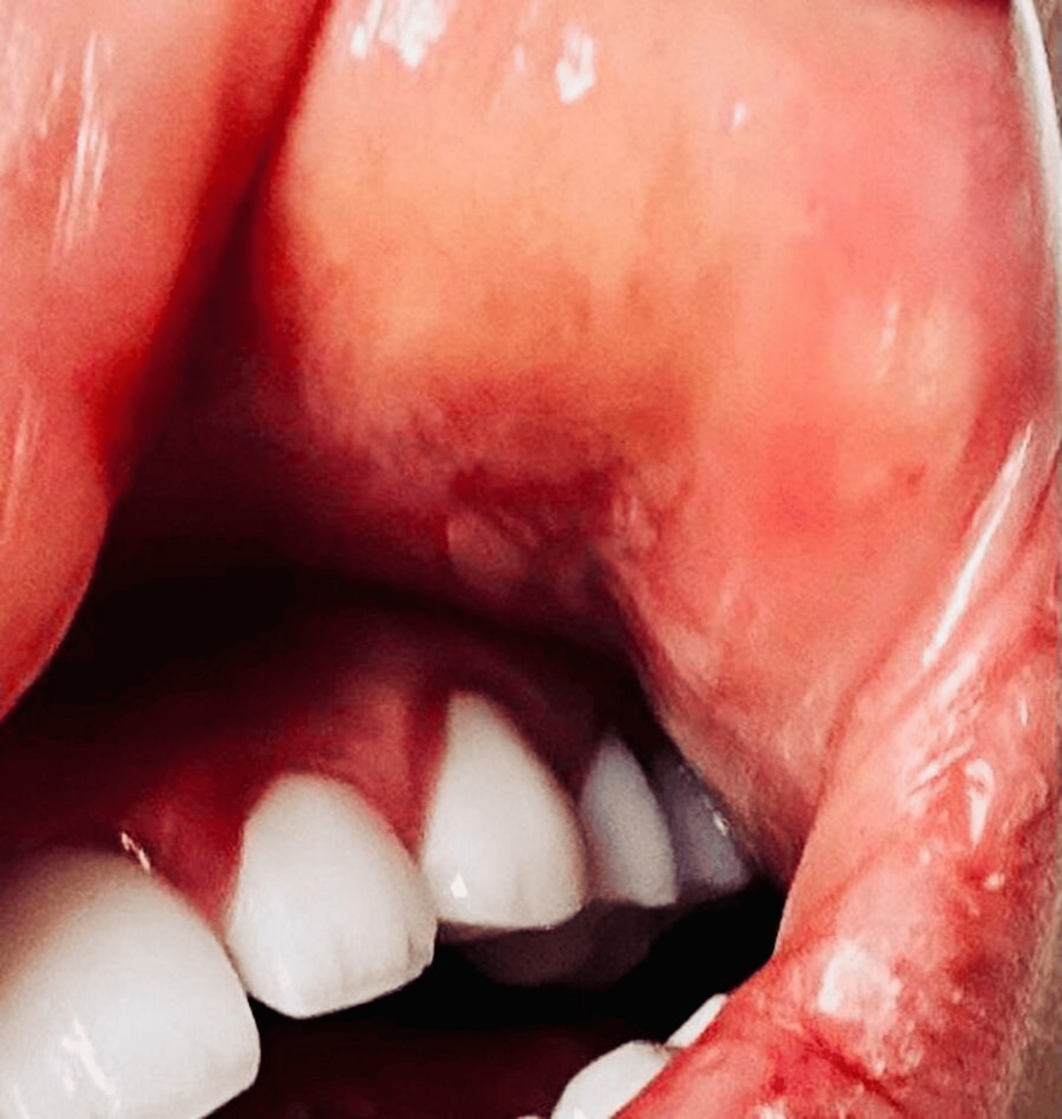
Oral ulcers as seen in Behçet disease

Following the diagnosis of pheochromocytoma, the patient developed painful genital ulcers and recurrent oral aphthous ulcers (Figure 2). These mucocutaneous lesions raised suspicion for BD, particularly given their classic distribution. While the initial presentation with pheochromocytoma is atypical, the subsequent development of hallmark symptoms and a positive HLA-B51 test - an allele associated with increased susceptibility to Behçet’s - supported the diagnosis. Alternative causes of oral and genital ulcers, such as viral infections, inflammatory bowel disease, and reactive arthritis, were ruled out. Based on the International Criteria for Behçet’s Disease (ICBD), a diagnosis of BD was made.

She was subsequently started on colchicine 0.5 mg twice a day. Since she had a slight improvement on alpha-blockers and her urinary retention persisted, with metanephrines and VMA trending upwards, the decision for surgical management of the adrenal tumor was made.

**Figure 3:**
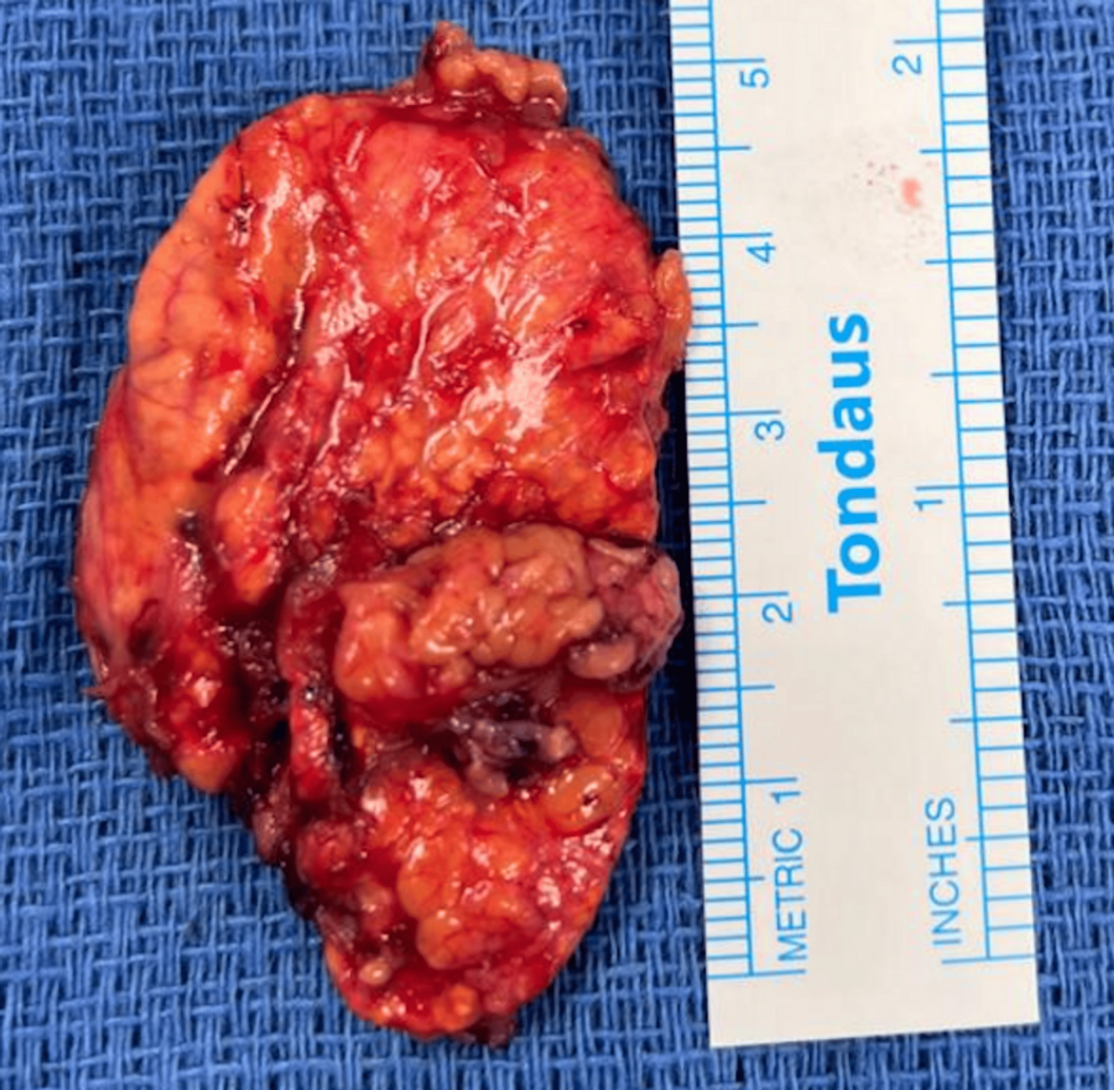
Surgical specimen of the resected left adrenal adenoma

**Figure 4:**
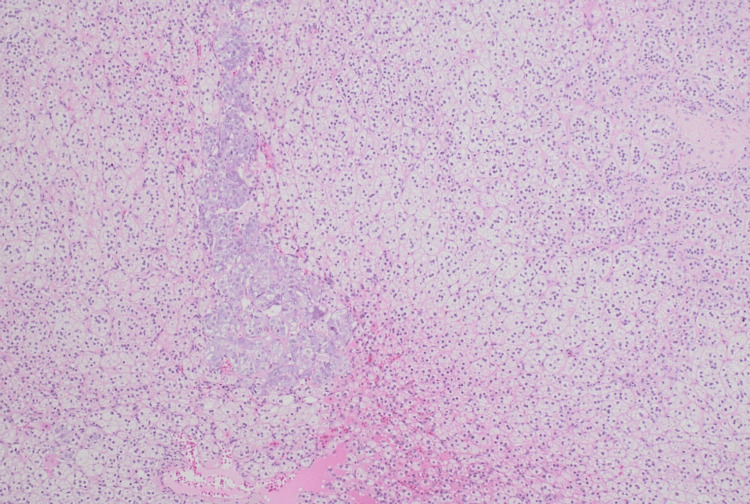
Hematoxylin & eosin, 4× microphotograph showing the hyperplastic adrenal cortex and preserved medulla

Given the biochemical and radiologic evidence, she underwent a laparoscopic left adrenalectomy. Intraoperatively, the adrenal gland was completely excised from the upper pole of the left kidney (Figure 3) and sent for histopathological examination, which confirmed adrenal cortical hyperplasia. However, the adrenal medulla was preserved and unaffected, which is atypical for pheochromocytoma (Figure 4).

Following surgery, the patient reported significant improvement in her systemic symptoms, including resolution of palpitations, dizziness, and urinary retention. Fifteen days post-surgery, her biochemical markers showed improvement: the repeat 24-hour urine metanephrine level was 47 µg (reference range: 36-209 µg/24 hr), normetanephrine was 206 µg (reference range: 131-612 µg/24 hr), and VMA was 1.5 mg (reference: <7 mg/24 hr). However, she subsequently presented with new-onset urinary incontinence. She also showed symptoms of low mood, for which a psychiatric evaluation was done, and she was started on sertraline 50 mg.

She was initiated on Betmiga (mirabegron) 50 mg for urinary incontinence and advised pelvic floor rehabilitation and bladder training. Urology follow-up recommended further evaluation with urodynamic studies. Forty-five days postoperatively, her 24-hour urine metanephrine level was 75 µg (reference: 36-209 µg/24 hr), normetanephrine was 386 µg (reference: 131-612 µg/24 hr), and VMA was 2 mg/24 hr (reference: <7 mg/24 hr), all of which had normalized. While all symptoms related to pheochromocytoma had resolved, urinary incontinence persisted. She remains under follow-up in the Rheumatology clinic for BD and continues to be managed by Urology for her persistent urinary symptoms.

## Discussion

Pheochromocytomas are rare catecholamine-secreting tumors originating from adrenal medullary chromaffin cells, often referred to as “the great mimicker” due to their wide array of nonspecific symptoms. While the classic triad includes headache, palpitations, and diaphoresis, patients may also present with more generalized features, such as episodic hypertension, anxiety, and cardiac-like symptoms, which complicate timely diagnosis. In the present case, the patient experienced recurrent palpitations and labile blood pressure, prompting extensive cardiovascular evaluations that initially yielded inconclusive results. Definitive diagnosis was eventually established through markedly elevated urinary metanephrines and VMA, along with CT imaging that revealed a left adrenal mass. Although additional diagnostic tools, such as the clonidine suppression test and DOTA PET scan, could have provided further support, these were either not performed or inaccessible due to insurance limitations. Imaging modalities remain critical for tumor localization and assessment of potential invasion or metastasis; magnetic resonance imaging (MRI) and functional imaging techniques, like metaiodobenzylguanidine (MIBG) or fluorodeoxyglucose positron emission tomography (FDG-PET), are especially useful in ambiguous cases [2].

Concurrent﻿ly, BD is a chronic, relapsing, multisystem inflammatory disorder, classically marked by recurrent oral and genital ulcers, uveitis, and systemic manifestations ranging from vascular to neurological involvement. Although its precise etiology remains elusive, BD is widely regarded as an autoimmune vasculitis, with a strong association to the HLA-B51 allele. Notably, recent evidence has demonstrated an elevated risk of malignancies among BD patients, including solid tumors such as renal and pulmonary carcinomas [4]. This increased oncologic susceptibility is believed to stem from persistent systemic inflammation, immune dysregulation, and prolonged use of immunosuppressive therapies. 

Based on the ICBD, our patient fulfills the diagnostic threshold with a total score of 5 points. The scoring includes 2 points for recurrent oral ulcers, 2 points for painful genital ulcers, and 1 point for vascular involvement - specifically, a documented history of DVT. There was no mention of skin lesions, ocular involvement, or neurological manifestations, and a pathergy test was not performed. According to the ICBD, a score of ≥4 confirms the diagnosis of BD. Thus, our patient meets the criteria with a cumulative score of 5 [5].

Although BD and pheochromocytoma are rarely reported together, emerging evidence suggests that pheochromocytomas may influence immune homeostasis in ways that could unmask or exacerbate autoimmune phenomena. While a direct causal relationship remains speculative, the interplay between chronic inflammation, immune dysregulation, and tumorigenesis warrants further exploration. As catecholamine-secreting tumors, they are known primarily for their cardiovascular and metabolic effects, but there is increasing recognition of their potential impact on the immune system [6].

Surgical resection remains the definitive treatment for pheochromocytoma and is typically curative [1]. The patient underwent a laparoscopic left adrenalectomy, and histopathology confirmed adrenal cortical hyperplasia; however, the adrenal medulla was preserved and unaffected, which is atypical for pheochromocytoma. Postoperatively, her cardiovascular and systemic symptoms improved significantly.

However, the subsequent emergence of depressive symptoms, urinary incontinence, and mucocutaneous lesions - including painful genital and oral ulcers - prompted further evaluation. The positive HLA-B51 test supported the diagnosis of BD [7].

In this case, our patient presented with both urge urinary incontinence and severe urinary retention - two contrasting symptoms that are rarely seen together. While urinary incontinence may be associated with BD and is more commonly observed in young patients with bladder involvement, urinary retention is not typical [8]. Conversely, acute urinary retention may be explained by the presence of a catecholamine-secreting pheochromocytoma, which leads to excessive alpha-adrenergic receptor stimulation. Specifically, overactivation of alpha-1 receptors present in the smooth muscle of the bladder neck and proximal urethra results in increased muscle tone and functional obstruction of the bladder outlet [9]. This sympathetic overdrive can impair bladder emptying, necessitating catheterization. 

Kim and Lee reported a rapid resolution of Behçet’s symptoms following the excision of a pheochromocytoma, proposing a possible immunoregulatory role of catecholamines in BD pathogenesis [10]. In a report by Lin et al., normalization of systemic lupus erythematosus (SLE)-associated autoantibodies following surgical removal of a pheochromocytoma suggests that the tumor may have contributed to immune dysregulation. This suggests that pheochromocytoma may have broader effects on the immune system, potentially influencing the development or severity of immune-mediated diseases like Behçet’s [11].

These findings suggest that neuroendocrine tumors may influence immune responses, particularly via excessive catecholamine secretion [12]. Chronic catecholamine exposure could alter cytokine profiles, modulate T-cell activity, and impair vascular integrity - mechanisms relevant to BD pathogenesis [13]. While definitive mechanistic links remain speculative, these cases highlight the need for further investigation into the immunomodulatory effects of pheochromocytoma and their potential role in triggering or exacerbating autoimmune disease. 

In summary, this report emphasizes the importance of a comprehensive and integrated diagnostic approach, highlighting how multidisciplinary care, meticulous preoperative preparation, and tailored postoperative immunomodulation are key to achieving favorable outcomes in patients with such rare and overlapping clinical entities.

## Conclusions

This case highlights a rare and medically significant overlap between pheochromocytoma and BD, underscoring the complex interplay between malignancy, immune dysregulation, and systemic inflammation. The clinical presentation, marked by the paradoxical coexistence of urinary retention attributed to pheochromocytoma and urinary incontinence associated with BD, challenges conventional diagnostic reasoning and emphasizes the need to consider overlapping multisystem disorders when symptoms appear contradictory. It also illustrates the potential immunomodulatory role of catecholamines in autoimmune processes, suggesting that the coexistence of these two conditions may not be coincidental. Clinically, this case reinforces the necessity of including pheochromocytoma in the differential diagnosis of patients with nonspecific systemic symptoms, such as anxiety, palpitations, and labile blood pressure, especially when initial evaluations yield inconclusive results. The simultaneous emergence or exacerbation of BD further complicates the clinical picture and requires heightened diagnostic vigilance. 

In such complex scenarios, maintaining a broad differential diagnosis is critical, particularly when new systemic symptoms arise in patients with established autoimmune conditions. A high index of suspicion for secondary causes of hypertension and atypical neurological or cardiovascular signs is essential. Most importantly, interdisciplinary collaboration is vital - balancing the immunosuppressive needs of autoimmune disease management with the perioperative risks of catecholamine-secreting tumors demands careful coordination.
